# National Trauma Registry Center, as a Backbone of Trauma Management and Research

**DOI:** 10.5812/atr.8487

**Published:** 2012-10-14

**Authors:** Mohammad Hossein Ebrahimzadeh

**Affiliations:** 1 Trauma Research Center, Mashhad University of Medical Sciences, Mashhad, IR Iran

**Keywords:** Wound and Injury, Trauma Management, Research

We are living in an era of growing incidence of trauma, violence and disasters around the world. In addition to raising of high velocity of vehicle accident injuries, we encounter many environmental disasters and military conflicts in the Middle East and Asia region. Currently, road traffic trauma is the 6th most common cause of death in developing countries and according to the WHO (World Health Organization) it will be placed at the 3rd leading cause of mortality and morbidity by 2030 (1, 2). While the mortality rate caused by trauma is almost doubled in our region compared to Western countries, disability from injury is much more common in comparison (3). According to the reports of the World Health Organization, among fatality causes in Iran, the road accidents is ranked the second cause of death and cover 11% of fatalities and 16% of years of life lost (YLL) due to a sudden death (4, 5). This is the tip of the iceberg of trauma, and there are a number of problems which are related to injury. There are various kinds of occupational, household, sport, assault, contact etc. injuries, which we have only scattered the data published occasionally and based on available cases and with very different background of the authors. There are also problems in transportation and admission of the cases to appropriate hospitals with supporting services that could manage these specific groups of the patients. Another significant problem is the overall condition of the referral centers considering the availability of modern equipment and skilled personnel. The evaluation of early and late complications of the injured patients and their final outcomes at the time of discharge and even after a while is another deficiency of our health care service for the injured people. Adding to this complexity there are some specific cultural and perhaps environmental types of injuries that are not common in other societies. The need for special consideration to the aforementioned problems as well as organizing and objectively oriented domestic researches in the fields of epidemiology management, estimation of disabilities, mortalities and burden on the economy of the country is now obviously evident more than any other times. Basically, it seems that the presence of an academic national registry center as an infrastructure vital coordinating center to all data related to trauma and its consequences from different organizations including police, fire fighting, services, emergency medical services, health care service system and forensic medicine organization. Such a trauma registry center can provide applicable information for health care administrators, health education authorities, insurance companies, clinicians and researchers which are involved in handling of different aspects of trauma. The pre-requisite of such a registry center foundation in Iran is to settle a national campaign for arrangement of both legislative and executive supports.
